# Functional Deficiency of MHC Class I Enhances LTP and Abolishes LTD in the Nucleus Accumbens of Mice

**DOI:** 10.1371/journal.pone.0107099

**Published:** 2014-09-30

**Authors:** Mitsuhiro Edamura, Gen Murakami, Hongrui Meng, Makoto Itakura, Ryuichi Shigemoto, Atsuo Fukuda, Daiichiro Nakahara

**Affiliations:** 1 Division of Psychology and Behavioral Neuroscience, Department of Integrated Human Sciences, Hamamatsu University School of Medicine, Higashi-ku, Hamamatsu, Japan; 2 Department of Biochemistry, Kitasato University School of Medicine, Sagamihara, Kanagawa, Japan; 3 Division of Cerebral Structure, National Institute for Physiological Sciences, Myodaiji, Okazaki, Japan; 4 Institute of Science and Technology Austria, Klosterneuburg, Austria; 5 Department of Neurophysiology, Hamamatsu University School of Medicine, Higashi-ku, Hamamatsu, Japan; Centre de Recherche Public de la Santé (CRP-Santé), Luxembourg

## Abstract

Major histocompatibility complex class I (MHCI) molecules were recently identified as novel regulators of synaptic plasticity. These molecules are expressed in various brain areas, especially in regions undergoing activity-dependent synaptic plasticity, but their role in the nucleus accumbens (NAc) is unknown. In this study, we investigated the effects of genetic disruption of MHCI function, through deletion of β2-microblobulin, which causes lack of cell surface expression of MHCI. First, we confirmed that MHCI molecules are expressed in the NAc core in wild-type mice. Second, we performed electrophysiological recordings with NAc core slices from wild-type and β2-microglobulin knock-out mice lacking cell surface expression of MHCI. We found that low frequency stimulation induced long-term depression in wild-type but not knock-out mice, whereas high frequency stimulation induced long-term potentiation in both genotypes, with a larger magnitude in knock-out mice. Furthermore, we demonstrated that knock-out mice showed more persistent behavioral sensitization to cocaine, which is a NAc-related behavior. Using this model, we analyzed the density of total AMPA receptors and their subunits GluR1 and GluR2 in the NAc core, by SDS-digested freeze-fracture replica labeling. After repeated cocaine exposure, the density of GluR1 was increased, but there was no change in total AMPA receptors and GluR2 levels in wild-type mice. In contrast, following repeated cocaine exposure, increased densities of total AMPA receptors, GluR1 and GluR2 were observed in knock-out mice. These results indicate that functional deficiency of MHCI enhances synaptic potentiation, induced by electrical and pharmacological stimulation.

## Introduction

Major histocompatibility complex class I (MHCI) molecules consist of a heavy chain (α1–α3) and a light chain, β2-microglobulin (β2m). MHCI proteins are expressed on the surface of most nucleated cells and are best known for their roles in cellular-mediated immunity. In the central nervous system, MHCI proteins are widely expressed and their expression level is particularly high in regions undergoing activity-dependent synaptic plasticity [1]–[6]. Recently, neuron–specific roles of MHCI have been examined and their important roles in synaptic plasticity have been reported, as described below.

By knocking-out both β2m and Tap1, a protein required for loading of peptides on MHCI, most cell-surface MHCI expression is eliminated [7]. In such double knock-out mice (β2m/Tap1^−/−^), the activity-dependent remodeling of retinal ganglion cell axons projecting to the developing lateral geniculate nucleus is impaired, and as a result, inappropriate projections that are normally eliminated during development persist [8]–[9]. Mice deficient in H2-K and H2-D, which are components of the heavy chains of MHCI, show a lower threshold for induction of long-term depression (LTD) in the cerebellum [10]. Furthermore, functional MHCI-deficient mice exhibit enhanced long-term potentiation (LTP) and absent LTD in the hippocampus [9], [11], [12]. These findings indicate that MHCI-deficient mice show abnormal synaptic plasticity in learning-related brain regions.

The nucleus accumbens (NAc) is involved in reward processing and reinforcement learning, and synaptic plasticity in this brain region has been extensively studied [13]–[19]. In slices prepared from the NAc core, electrical stimulation can induce LTP at high frequency [13], [14], [20] or LTD at low frequency [15], [16], [17]. On the other hand, the AMPA/NMDA receptor ratio in the NAc of animals treated repeatedly with cocaine is enhanced [18]. Likewise, the extracellular/intracellular ratio of GluR1 and GluR2 in the NAc of animals treated repeatedly with cocaine is enhanced [19], indicating enhanced synaptic connectivity. As such, synaptic plasticity can be induced in the NAc by both electrical and pharmacological stimuli. However, it remains unknown whether MHCI molecules are involved in these forms of synaptic plasticity in the NAc. As synaptic plasticity in the NAc closely relates to addictive behaviors like sensitization, craving and relapse [21]–[24], we examined this possibility in the present study.

First, we examined whether MHCI is expressed in the NAc of wild-type (WT) mice, by immunostaining methods. Second, to examine the involvement of MHCI in synaptic plasticity induced by electrical stimulation, we prepared NAc slices from β2m knock-out (β2m^−/−^) mice in which a majority of cell-surface MHCI expression is eliminated [7] and analyzed LTP and LTD by electrophysiological methods. Finally, to examine the involvement of MHCI in synaptic plasticity induced by pharmacological stimulation, we analyzed AMPA receptor density in the synapse directly by SDS-digested freeze-fracture replica labeling, using NAc samples prepared from WT and β2m^−/−^ mice treated repeatedly with cocaine.

## Materials and Methods

### Subjects

β2m^−/−^ mice (maintained on a C57BL/6J background) were purchased from a commercial supplier (Jackson Labs, Bar Harbor, ME, USA). WT and β2m^−/−^ mice were generated by heterozygous mutants, and we used WT littermates or WT C57Bl/6J mice (Nippon SLC, Inc., Shizuoka, Japan) as a control group for the β2m^−/−^ mice. All the mice were housed in specific pathogen-free (SPF) facility and behavioral analysis was also done there. In-house monitoring was performed every 3 months using a Monilisa IVA kit (Wakamoto Pharmaceutical Co., Ltd., Tokyo, Japan) that detects four major organisms, Sendai virus, mouse hepatitis virus, mycoplasma and Tyzzer’s organism [25]. No infections were detected in any of the rooms in which mice used in this study were maintained. Male mice were used in the present study. Mice were housed in groups of three to six animals until 2 weeks before the first experiment and then individually in standard laboratory Plexiglas cages under a 12-h light/12-h dark schedule (lights on at 07∶00, lights off at 19∶00), with free access to food and water. All experiments were performed during the light period, when the mice were 8–12 weeks of age.

### Ethics Statement

All procedures were approved by the Hamamatsu University School of Medicine Animal Care and Use Committee, and carried out in accordance with National Institute of Health general guidelines for the Care and Use of Laboratory animals (NIH Publications No. 86-23). All efforts were made to minimize animal suffering and the number of animals used.

### Immunohistochemical staining of MHCΙ

Mice were deeply anesthetized with diethyl ether and gently perfused transcardially with PBS followed by 4% paraformaldehyde. Then, whole brains were dissected and kept in the same solution at 4°C overnight. After cryoprotection with 30% sucrose in PBS solution, coronal slices at 60 µm thickness were prepared by a cryostat (HM550, Thermo Scientific, Waltham, MA, USA). MHCI was stained with mouse monoclonal antibody against MHCI (1/100, OX-18, AbD Serotec, Oxford, UK) in PBS with 3% BSA at 4°C overnight. For the negative control, the primary antibody was omitted. Then rabbit polyclonal antibody (1/500, AB5620, Millipore Bioscience Research Reagents, Temecula, CA, USA) was incubated for the staining of Neurogranin in PBS with 3% BSA and 0.5% Triton x-100 at room temperature for 1 hr. Those slices were labeled with anti-Rabbit Alexa fluor 488 (1/500, Life Technologies, Carlsbad, CA, USA) for staining of Neurogranin and anti-Mouse Alexa fluor 546 (1/500, Life Technologies) for staining of MHCΙ. After being stained, the slices were embedded in ProLong Gold antifade reagents (Invitrogen, Life Technologies) for observations. Images were taken by a confocal microscope (FV1000-D, Olympus, Tokyo, Japan).

### Electrophysiology

WT and β2m^−/−^ mice were decapitated and their brains were quickly removed and submerged in a cutting solution (124 mM Sucrose, 22 mM NaHCO_3_, 5 mM KCl, 1.25 mM NaH_2_PO_4_, 2.5 mM CaCl_2_, 1 mM MgCl_2_, 10 mM D-Glucose), bubbled by 95% oxygen and 5% carbon dioxide over 20 minutes. Parasagittal slices (350 µm thickness) were prepared by a vibratome slicer (BVS-100, Bio Research Center, Nagoya, Japan) and trimmed to include the NAc and prefrontal cortex. Slices were transferred to a recovery chamber filled with artificial cerebrospinal fluid (ACSF) (124 mM NaCl, 22 mM NaHCO_3_, 5 mM KCl, 1.25 mM NaHPO_4_, 2.5 mM CaCl_2_, 1 mM MgCl_2_, 10 mM D-Glucose) that was continuously bubbled by 95% oxygen and 5% carbon dioxide, and recovered over 1 h at room temperature (25–29°C). Then, the slices were transferred to the recording dish in a multi electrode array system (MEA60, Multi Channel Systems, Reutlingen, Germany) and constantly perfused by bubbled ACSF containing 50 µM picrotoxin at 37°C. The recording dish in the multi electrode array system had 60 titanium nitride electrodes, a 30 µm diameter at its base, and was arranged in 8×8 matrix with a 200 µm inter-electrode interval. Using these electrodes, we stimulated the tissue electrically and recorded extracellular potentials [26]–[29]. The multi-electrode array was perforated between the electrodes to provide for suctioning the slice to the bottom and thereby enabling stable recording over several hours [30]. After at least 20 minutes of perfusion, 0–130 µA biphasic pulses (100 µs each) with a 10 µA step were applied to evoke field excitatory postsynaptic potentials (fEPSPs) and I/V curves were measured. The stimulation intensity evoking a half-maximal response was used for the paired-pulse ratio (PPR) measurements and single pulse stimulations. Electrical stimulation was applied at the NAc core, 0–400 µm from the border between the cortex and the NAc core, to stimulate primarily infralimbic and prelimbic afferents [13], and fEPSPs were recorded from a neighboring electrode, 200 µm caudal to the stimulation electrode. Recording was performed using commercial software (MC_Rack; Multi Channel Systems) with a 25 kHz sampling rate. For PPR measurements, two pulses with 30, 50 or 100 ms inter pulse interval (ISI) were applied. We successively recorded fEPSPs by applying single pulses every 30 seconds over at least 10 minutes. Then, we applied tetanic stimulation to the tissue and recorded fEPSPs for another 50 minutes. We applied high frequency stimulation (HFS) at 100 Hz for 1 second, which provoked a 90% response in the I/V curve measurements; low frequency stimulation (LFS) at 10 Hz for 5 minutes, which provoked a 30% response; or stimulation at 1 Hz for 15 minutes, which provoked a 30% response. The fEPSP slope was analyzed by commercial analysis software (Peak Analysis; ADInstruments; Dunedin, New Zealand). The average fEPSP slope over the 5 minutes before tetanic stimulation was set to 100%, and used to compare to the average during the 45–50 minutes after stimulation.

### Cocaine treatment and locomotor activity

WT and β2m^−/−^ mice were given intraperitoneal injections of either saline (0.9% NaCl) or cocaine (20 mg/kg) at a volume of 10 ml/kg. Immediately after each injection, locomotor activity was measured for 60 min in eight identical activity monitors measuring 30×30 cm with pyroelectric infrared sensors attached on the lid (Biotex, Kyoto, Japan). It detects temperature changes caused by locomotion at time resolution of 200 ms and counts them up. After 3 days of saline injections, mice were divided into four groups that received seven daily injections of either saline or cocaine. The groups comprised WT mice treated with saline (WT-Sal) or cocaine (WT-Coc) and β2m^−/−^ mice treated with saline (β2m^−/−^-Sal) or cocaine (β2m^−/−^-Coc). Following 14 days of withdrawal, all groups received a challenge injection of cocaine (20 mg/kg) and locomotor activity was assessed.

### SDS-digested freeze-fracture replica labeling (SDS-FRL)

SDS-FRL can be used to observe the two dimensional structure of cell membranes and the distribution of membrane proteins [31]–[33]. Excitatory synapses were identified by the intramembrane particle (IMP) cluster on its exoplasmic face [34]. In this experiment, mice were given intraperitoneal injections of either saline or cocaine (20 mg/kg). After 3 days of saline injections, mice were divided into groups that received seven daily injections of either saline or cocaine with no measurement of locomotor activity. Following 14 days of withdrawal, mice were decapitated and their brains were removed. We measured the density of AMPA receptors on excitatory synapses in the NAc by SDS-FRL. Mice were anesthetized by pentobarbital (40 mg/kg, i.p.) and fixed with 2% paraformaldehyde by perfusion fixation. Coronal slices (150 µm thickness) were prepared by a micro slicer (Linearslicer PRO7, Dosaka, Kyoto, Japan) and the NAc core was trimmed. Trimmed sections were submerged in 0.1 M phosphate buffer with 30% glycerol at 4°C overnight, and the sections were then frozen quickly by a high-pressure freezing machine (HPM010, BAL-TEC, Balzers, Liechtenstein). The frozen slices were then freeze-fractured and replicated with carbon (5 nm), shadowed by platinum (2 nm), and replicated again with carbon (15–20 nm) in a freeze fracture machine (JFD-II, JEOL, Tokyo, Japan). After thawing, tissue debris attached to the replicas were digested with gentle stirring at 80°C for 16 hours in a solution of 15 mM Tris-HCl, 20% sucrose, and 2.5% sodium lauryl sulfate.

We used three different primary antibodies and performed single labeling on each separated replica. These antibodies were the pan-AMPA antibody raised in rabbit, which binds to GluR1, GluR2, GluR3 and GluR4 receptors (the specificity of this antibody was described in ref. [35]), a GluR1 antibody (rabbit polyclonal antibody; the specificity of this antibody was described in ref. [36]), and a GluR2 antibody (mouse monoclonal antibody, Millipore Bioscience Research Reagents). To eliminate possible errors between preparations, slices for comparison were replicated in parallel and immunolabeled at the same time, and protein densities were normalized for each control. In this way, we could compare the groups using equivalent procedures. The replicas were washed in washing buffer (0.1% Tween-20, 0.05% BSA, 0.05% NaN3, in TBS) and blocked for 30 minutes in another washing buffer in which the BSA concentration was increased to 5%. Replicas were reacted with the pan-AMPA (3 µg/ml), GluR1 (5 µg/ml), and GluR2 (15 µg/ml) antibodies at 15°C overnight with shaking, followed by incubation in an anti-rabbit (British Biocell International, Cardiff, UK) or anti-mouse (GE Healthcare, Buckinghamshire, UK) secondary antibody conjugated with 5 nm gold particles for 1 hour at 37°C. The replicas were examined with an electron microscope (JEM-1010, JEOL, Tokyo, Japan) and photographed at magnification of 100,000. Thirty synapses in each replica were photographed randomly and used for analysis. Immunogold particles were counted in excitatory postsynaptic areas indicated by the IMP cluster, which was defined by densely packed IMPs at a distance of <15 nm from each other [34], [37], [38]. The outline of the synaptic sites was demarcated freehand, and the areas of the synapses were measured by commercial analysis software (Digitalmicrograph, Gatan, Pleasanton, CA, USA). The density was calculated by dividing the number of immunogold particles counted by the total area of the synapse. Then the average density was calculated in each replica. The percent density was calculated as the average density in each replica divided by the average density from all replicas in the control group multiplied by 100.

### Statistical analysis

Data from the electrophysiology and SDS-FRL experiments were compared using the Student’s t-test, except for evaluating the establishment of LTP or LTD in which we used the Wilcoxon signed rank test. N = number of animals; n = number of slices. Locomotor sensitization to cocaine was assessed using two-way analysis of variance (ANOVA) with repeated measures. Locomotor responses to cocaine challenge in the four groups were compared using two-way ANOVA. Post-hoc comparisons were performed using the Bonferroni test. Statistical calculations were made using the statistical software SPSS (version 17.0) for Windows. The level of significance was set at p<0.05.

## Results

### MHCI molecules are expressed in accumbal neurons

**Figure 1:**
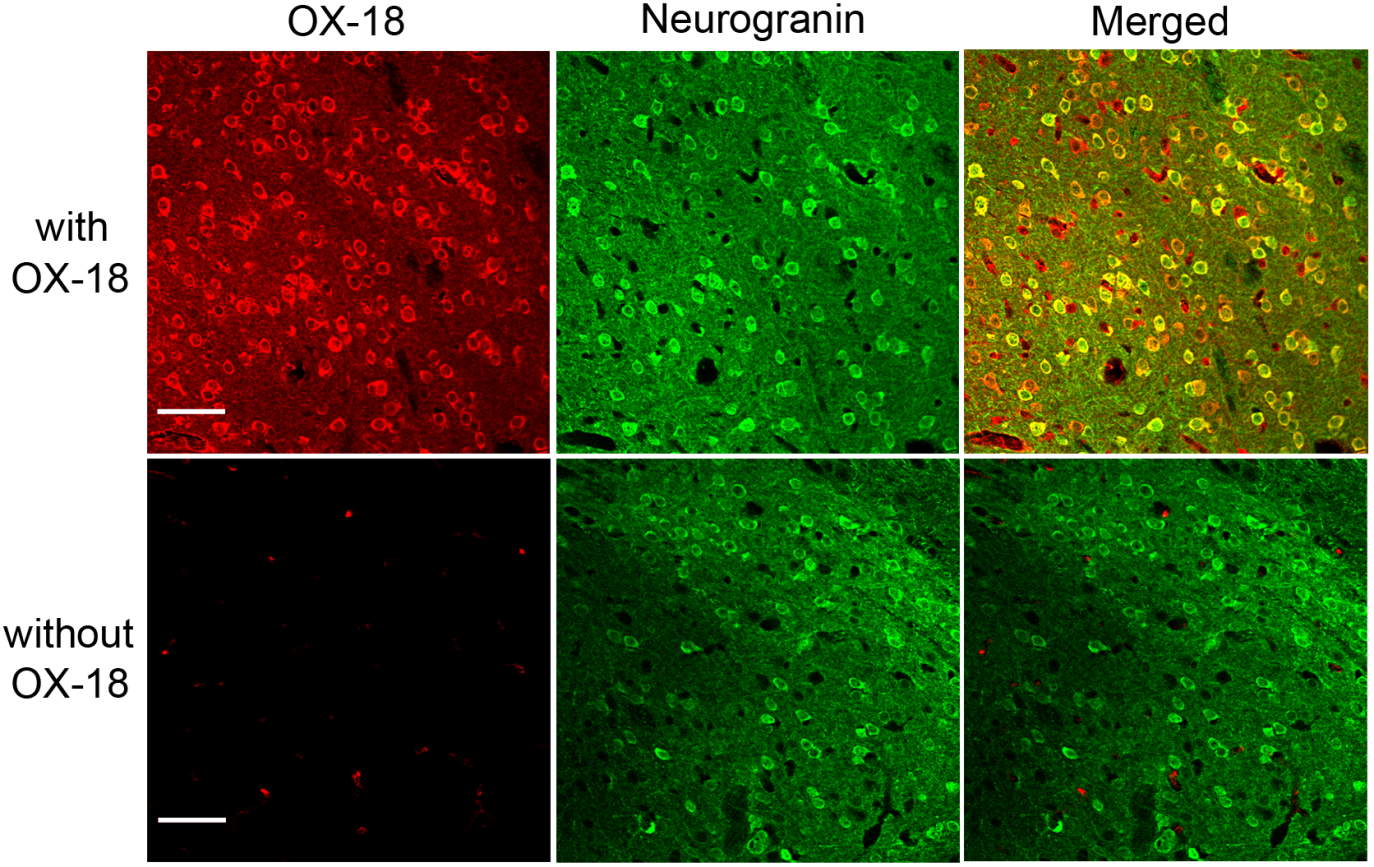
MHCI molecules are expressed in the nucleus accumbens core in WT mice. Staining for MHCI with OX-18 antibody (upper-left), Neurogranin (upper-center) and the merged image (upper-right) are shown. Lower images represent negative controls without OX-18. Scale bar represents 50 µm.

Recent studies revealed that MHCI molecules are expressed in numerous brain areas [2]. In this study, we stained slices containing NAc core with and without antibodies against MHCI in WT mice (Fig. 1). Double-staining with neurogranin, a marker of neurons, showed that MHCI molecules were expressed in neurons. No stained cell was found in the experiment without primary antibodies. This indicates that MHCI molecules exist in the NAc and could have a role in synaptic plasticity in this brain area.

### LTP is enhanced and LTD is abolished in the NAc of β2m^−/−^ mice

**Figure 2:**
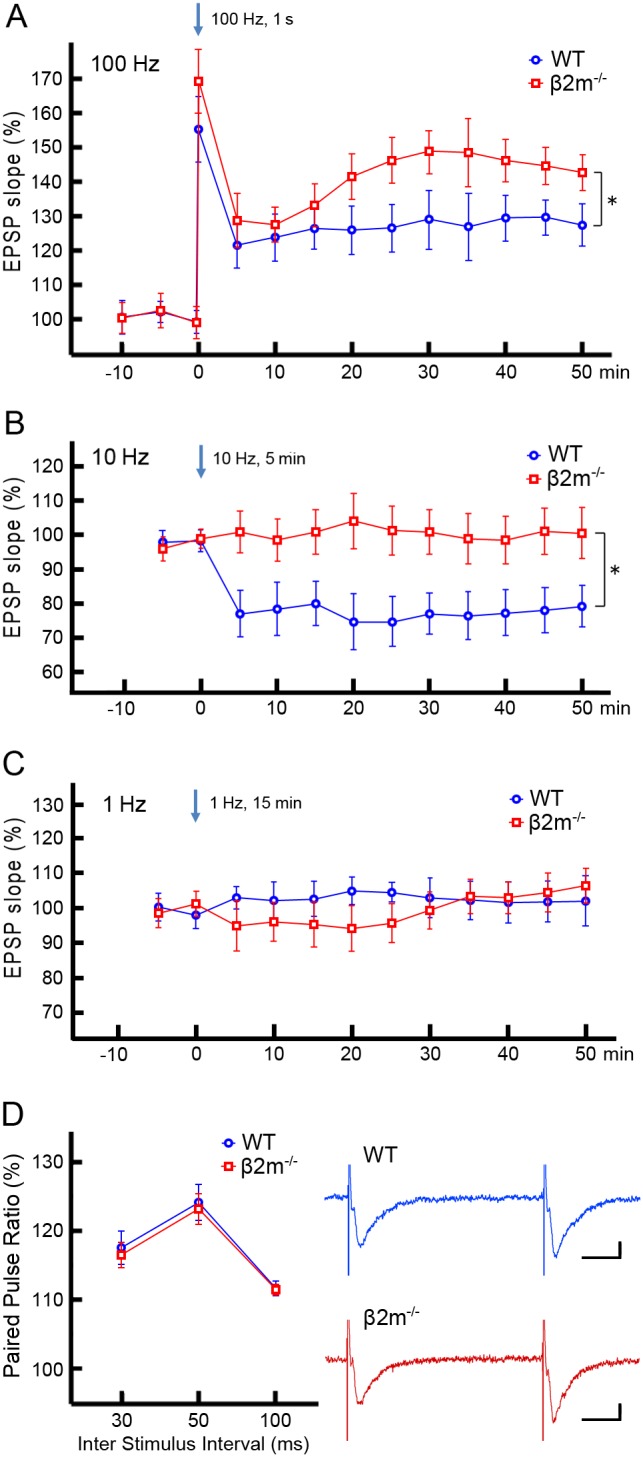
Comparison of the fEPSP slopes. A) Comparison between WT mice and β2m^−/−^ mice after high frequency stimulation (HFS) at 100 Hz. HFS induced LTP in both groups (WT: 125.8±4.5%, n = 9/N = 5, T = 1, p<0.01; β2m^−/−^: 141.8±5.7%, n = 11/N = 6, T = 0, p<0.001; Signed rank test), whereas LTP was enhanced in the β2m^−/−^ group (t(18) = 2.107, p<0.05; Student’s t-test). B) Comparison between WT and β2m^−/−^ mice after low frequency stimulation (LFS) at 10 Hz. LFS induced LTD in WT mice (79.2±5.9%, n = 9/N = 6, T = 1, p<0.05), whereas it was ineffective in β2m^−/−^ mice (103.2±7.3%, n = 9/N = 6, T = 18, p = 0.65). At 45–50 min after LFS, the fEPSP slope in β2m^−/−^ mice was significantly higher than in WT mice (t(16) = 2.548, p<0.05). C) Comparison between WT and β2m^−/−^ mice after 1 Hz stimulation. Neither LTP nor LTD was induced by this stimulation (WT: 103.4±7.1%, n = 7/N = 6, T = 9, p = 0.47; β2m^−/−^: 100.7±5.7%, n = 7/N = 6, T = 12, p = 0.81). There was no significant difference in the fEPSP slope at 45–50 min after 1 Hz/15 min stimulation between genotypes (t(12) = 0.302, p = 0.768). D) Comparison of paired pulse ratios (PPRs). (Left) PPRs in WT and β2m^−/−^ mice at 30, 50 and 100 ms inter stimulus interval (ISI). There was no significant difference in the PPRs of WT (ISI 30 ms: 117.9±2.4%; ISI 50 ms: 124.5±2.6%; ISI100 ms: 111.7±1.0%) (n = 9/N = 5) and β2m^−/−^ (ISI 30 ms: 116.8±1.9%; ISI 50 ms: 123.2±2.3%; ISI 100 ms: 111.5±0.8%) (n = 9/N = 5) mice, in all tested ISIs (ISI 30 ms: t(16) = 0.349, p = 0.731; ISI 50 ms: t(16) = 0.355, p = 0.727; ISI 100 ms: t(16) = 0.164, p = 0.872). (Right) Representative traces of fEPSPs at 50 ms inter stimulus interval (blue: WT; red: β2m^−/−^). Vertical scale bars represent 100 µV, and horizontal scale bars represent 10 ms. *p<0.05. n = slices/N = animals.

Synaptic plasticity in the NAc is dependent on the stimulation frequency, with high frequencies producing LTP [13], [39] and low frequencies producing LTD [15], [17]. We therefore examined the effects of three different stimulation frequencies on accumbal synaptic plasticity. In WT mice, HFS at 100 Hz for 1 sec resulted in an increase in the slope of the fEPSP at 45–50 min after tetanus (Fig. 2A: 125.8±4.5% of pretetanus baseline, n = 9/N = 5, T = 1, p<0.01; Signed rank test). In contrast, low frequency stimulation (LFS) at 10 Hz for 5 min induced a significant decrease in the slope of the fEPSP at 45–50 min after tetanus (Fig. 2B: 79.2±5.9% of baseline, n = 9/N = 6, T = 1, p<0.05). There was no significant change in the fEPSP slope upon 1 Hz stimulation for 15 min at 45–50 min after tetanus (Fig. 2C: 103.4±7.1% of baseline, n = 7/N = 6, T = 9, p = 0.47). Thus, we confirmed previous findings [16] demonstrating that electric stimulation induces LTD at 10 Hz as well as LTP at 100 Hz in the NAc.

Next, we tested whether β2m contributes to LTP and/or LTD at accumbal synapses. As in WT mice, we examined the effects of three different stimulation frequencies on synaptic plasticity in β2m^−/−^ accumbal slices. HFS at 100 Hz resulted in a significant increase in the slope of the fEPSP curve at 45–50 min after tetanus (Fig. 2A: 141.8±5.7% of pretetanus baseline, n = 11/N = 6, T = 0, p<0.001). This increase was significantly larger than that in WT mice (t(18) = 2.107, p<0.05; Student’s t-test). However, LFS at 10 Hz did not elicit LTD (Fig. 2B: 103.2±7.3% of baseline, n = 9/N = 6, T = 18, p = 0.65). At 45–50 min after LFS, the fEPSP slope in β2m^−/−^ mice was significantly higher than in WT mice (t(16) = 2.548, p<0.05). Additionally, there was no significant change in the fEPSP slope upon 1 Hz stimulation (Fig. 2C: 100.7±5.7% of baseline, n = 7/N = 6, T = 12, p = 0.81). We also examined the paired pulse ratio (PPR), which reflects the function of presynaptic glutamate release, in mice of both genotypes (Fig. 2D). There was no significant difference in the PPRs of wild-type and β2m^−/−^ mice, in all of inter-pulse intervals including 30 msec (WT 117.9±2.4%, n = 9/N = 5; β2m^−/−^ 116.8±1.9%, n = 9/N = 5; t(16) = 0.349, p = 0.731), 50 msec (WT 124.5±2.6%, n = 9/N = 5; β2m^−/−^ 123.2±2.3%, n = 9/N = 5; t(16) = 0.355, p = 0.727) and 100 msec (WT 111.7±1.0%, n = 9/N = 5; β2m^−/−^ 111.5±0.8%, n = 9/N = 5; t(16) = 0.164, p = 0.872). This suggests that enhanced LTP we observed in β2m^−/−^ mice is unlikely to result from altered probability of glutamate release.

### Behavioral sensitization to cocaine is augmented in β2m^−/−^ mice

**Figure 3:**
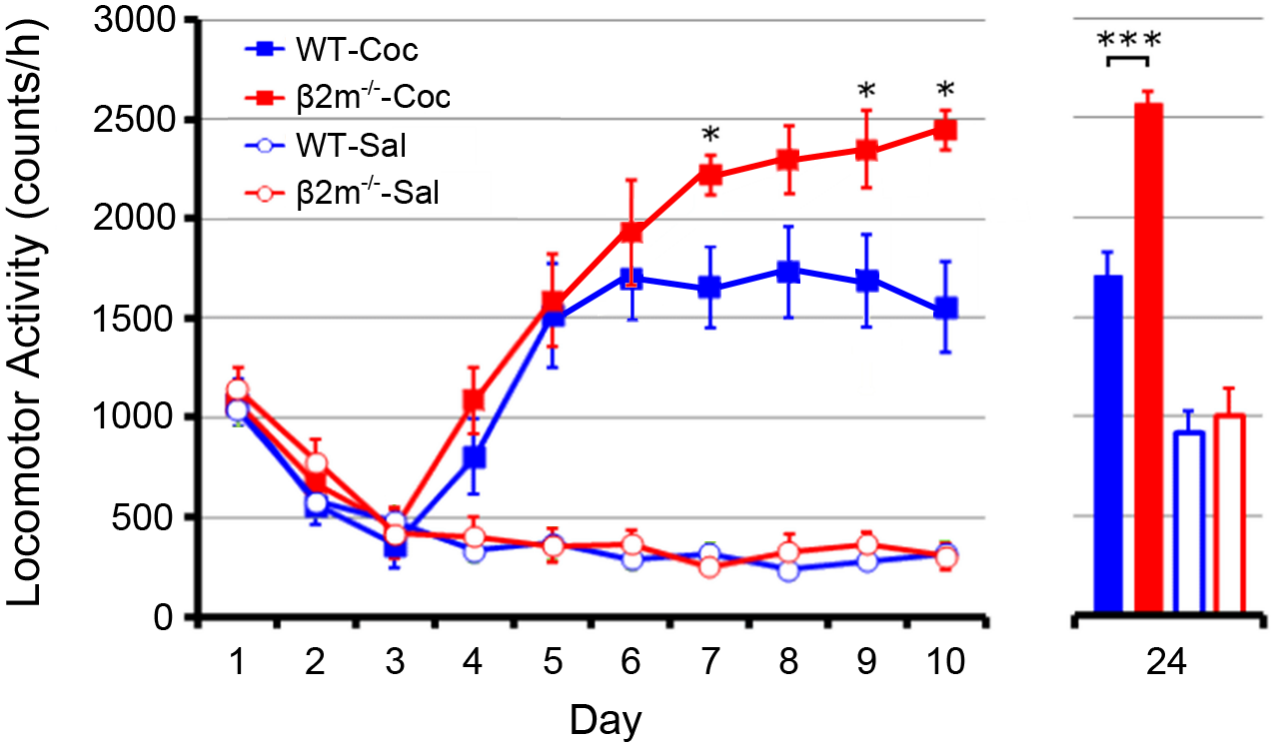
Behavioral sensitization elicited by repeated cocaine exposure. After three days of saline injections, WT and β2m^−/−^ mice were divided into groups that received daily injections of saline or cocaine (20 mg/kg) for 7 days. These groups were composed of WT mice treated with saline (WT-Sal) (n = 6) or cocaine (WT-Coc) (n = 6) and β2m^−/−^ mice treated with saline (β2m^−/−^-Sal) (n = 6) or cocaine (β2m^−/−^-Coc) (n = 6). Mice of both genotypes showed increased locomotor activity following cocaine but not saline injections. Analysis was conducted over a 8-day period from day 3 to day 10 in WT-Coc and β2m^−/−^-Coc groups. Two-way ANOVA with repeated measures revealed significant interactions of day × genotype (F(7, 70) = 2.892, p<0.05), a main effect of day (F(7, 70) = 42.285, P<0.001) and genotype (F(1, 10) = 5.027, p<0.05). Bonferroni post-hoc test revealed significant differences at day 7, day 9 and day 10. Furthermore, the β2m^−/−^-Coc group displayed a larger response to a challenge dose of cocaine after withdrawal (Day 24) than the WT-Coc group. Two-way ANOVA revealed a main effect of genotype (F(1, 20) = 19.674, p<0.001) and treatment (F(1, 20) = 121,488, p<0.001), and a significant interaction of genotype × treatment (F(1, 20) = 13.138, p<0.01). A significant difference was observed between genotypes treated with cocaine (p<0.001, Bonferroni post-hoc test). *p<0.05 β2m^−/−^-Coc versus WT-Coc; ***p<0.001 β2m^−/−^-Coc versus WT-Coc.

To induce behavioral sensitization, we gave repeated cocaine injections to WT and β2m^−/−^ mice. After 3 days of saline injections to acclimatize animals to the activity test box, acute locomotor responses to the drug increased dramatically across multiple days of testing in both WT (activity count: day 4-saline 329.8±56.9, n = 6; day 4-cocaine 804.2±188.5, n = 6; day 10-saline 317.3±57.3, n = 6; day 10-cocaine 1555.2±224.1, n = 6) and β2m^−/−^ mice (activity count: day 4-saline 405.2±96.9, n = 6; day 4-cocaine 1087.7±168.5, n = 6; day 10-saline 304.5±67.1, n = 6; day 10-cocaine 2443.2±96.6, n = 6) (Fig. 3). Furthermore, β2m^−/−^ mice showed a much larger increase in locomotor sensitization to cocaine than WT mice (p<0.05). To test whether this method produced long-lasting sensitization, we administered a challenge dose of cocaine (20 mg/kg) to both saline- and cocaine-treated animals of WT and β2m^−/−^ groups followed by a 14-day withdrawal. Mice pretreated with chronic cocaine exhibited a much greater locomotor response to the drug than did saline-treated animals in both WT (activity count: saline 917±109.3, n = 6; cocaine 1707±112.2, n = 6, p<0.001) and β2m^−/−^ mice (activity count: saline 1003.7±132.7, n = 6; cocaine, 2569.2±59.5, n = 6, p<0.001) (Fig. 3). Again, this sensitized response in β2m^−/−^ mice was larger than in WT animals (p<0.001). These findings indicate that behavioral sensitization after 7 days of exposure to cocaine is enhanced in β2m^−/−^ mice compared with WT mice, and this sensitization lasts for at least 2 weeks.

### Synaptic GluR1 is increased in cocaine-treated WT mice whereas pan-AMPA, GluR1 and GluR2 are increased in cocaine-treated β2m^−/−^ mice

**Figure 4:**
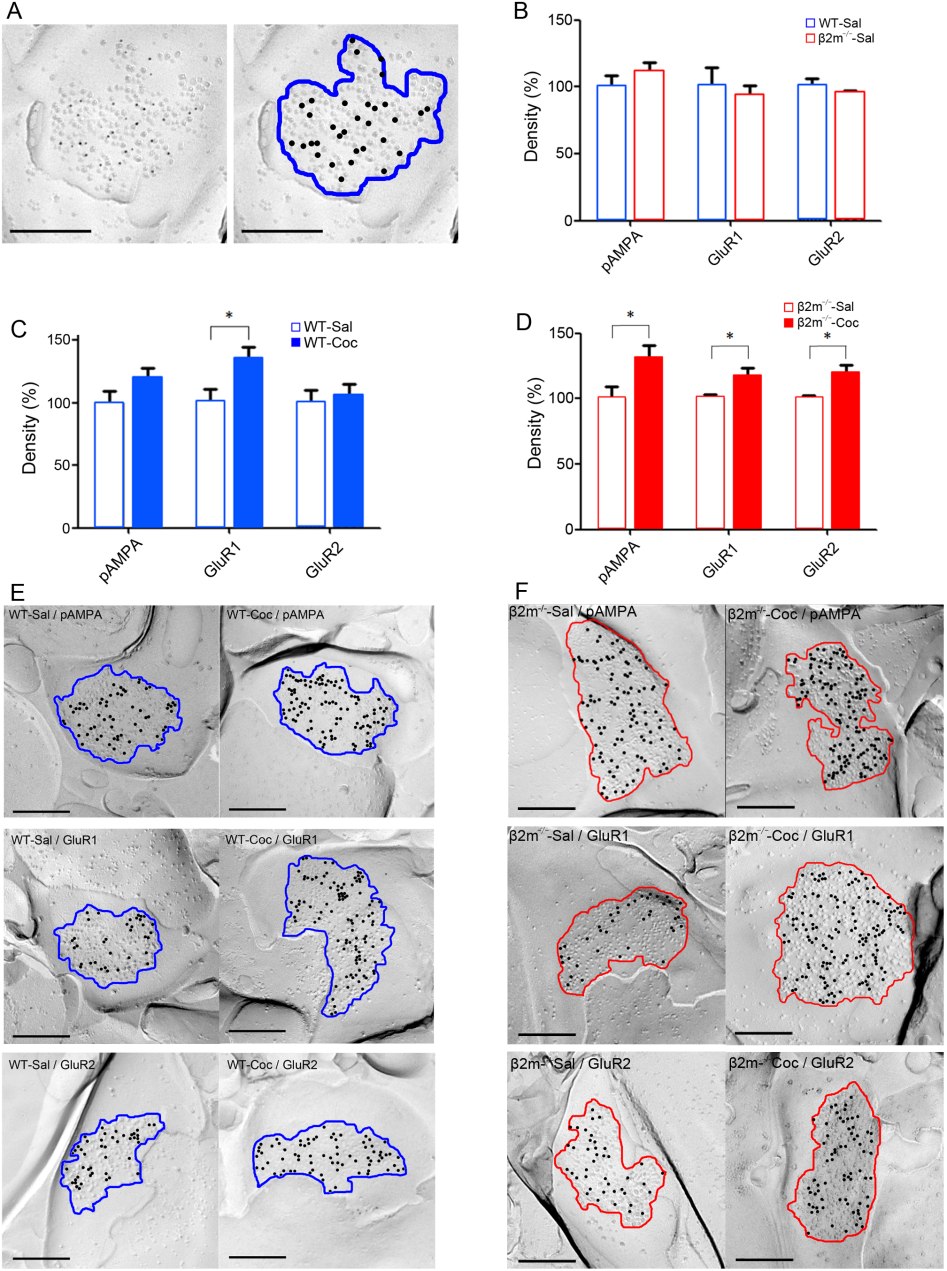
Comparison of AMPA receptor densities. A) A raw electron micrograph image of SDS-FRL replica immunolabeled with pan-AMPA antibody (left), and an analyzed image (right). A representative replica from a wild type mouse treated with saline (WT-Sal) is shown. The blue area indicates intra-membrane particle clusters and the black dots indicate immunogold particles. B) Densities of pan-AMPA, GluR1, and GluR2 in WT-Sal mice and β2m mice treated with saline (β2m^−/−^-Sal). The densities normalized to WT in each receptor were: pan-AMPA: β2m^−/−^-Sal, 110.0±8.5%; GluR1: β2m^−/−^-Sal, 92.9±7.8%; GluR2: β2m^−/−^-Sal, 95.4±1.5%, n = 6 [for each receptor 6 replicas from three animals, 30 synapses per replica]. C) Comparison of pan-AMPA, GluR1 and GluR2 densities between WT-Sal mice and wild type mice treated with cocaine (WT-Coc). The densities normalized to saline group in each receptor were: pan-AMPA: WT-Coc, 119.4±8.3%; GluR1: WT–Coc, 135.3±8.9%; GluR2: WT–Coc, 105.6±9.6%, n = 6 [for each receptor six replicas from three animals, 30 synapses per replica]. D) Comparison of pan-AMPA, GluR1 and GluR2 densities between β2m^−/−^-Sal mice and β2m^−/−^ mice treated with cocaine (β2m^−/−^-Coc). The densities normalized to saline group in each receptor were: pan-AMPA: β2m^−/−^-Coc, 130.9±10.0%; GluR1: β2m^−/−^-Coc, 117.5±5.8%; GluR2: β2m^−/−^-Coc, 119.7±6.2%, n = 6 [for each receptor six replicas from three animals, 30 synapses per replica]. E) Representative electron micrographs of the replicas from WT-Sal and WT-Coc mice. The blue area indicates intra-membrane particle clusters and the black dots indicate immunogold particles. F) Representative electron micrographs of the replicas from β2m^−/−^-Sal and β2m^−/−^-Coc mice. The red area indicates intra-membrane particle clusters and the black dots indicate immunogold particles. Scale bars represent 200 nm. *p<0.05.

Chronic cocaine administration elicits long-lasting alteration of synaptic excitatory transmission in the NAc, likely contributing to behavioral sensitization [40]. Therefore, to assess the impact of genetic deletion of β2m on synaptic excitatory transmission, we used SDS-FRL to measure the density of AMPA receptors and their subunits in the NAc core after repeated treatment with either cocaine or saline for 7 days. To minimize possible differences between preparations, we only compared the samples that were immunolabeled at the same time. First, we compared expression of pan-AMPA, GluR1, and GluR2 immunolabeling between saline-treated WT (Fig. 4A) and β2m^−/−^ mice, and confirmed that there was no difference in the basal expression of AMPA receptors between these genotypes (Fig. 4B). The densities normalized to WT in each receptor were: pan-AMPA: β2m^−/−^-Sal, 110.0±8.5%; GluR1: β2m^−/−^-Sal, 92.9±7.8%; GluR2: β2m^−/−^-Sal, 95.4±1.5%; n = 6 [for each receptor 6 replicas from three animals, 30 synapses per replica] (pan-AMPA: t(10) = 0.82, p = 0.43; GluR1: t(10) = 0.44, p = 0.67; GluR2: t(10) = 0.69, p = 0.51; Student’s t-test). Next, we analyzed the effects of repeated cocaine exposure on AMPA receptor expression in the NAc of WT mice (Figs. 4C, 4E). The densities normalized to saline in each receptor were: pan-AMPA: WT-Coc, 119.4±8.3%; GluR1: WT–Coc, 135.3±8.9%; GluR2: WT–Coc, 105.6±9.6%; n = 6 [for each receptor 6 replicas from three animals, 30 synapses per replica]. Fourteen days after the final cocaine administration, synapses in WT mice showed a significant increase in the density of GluR1 (GluR1: t(10) = 2.49, p<0.05) compared with saline administration. In pan-AMPA, a moderate increase was observed, but it was not statistically significant (t(10) = 1.52, p = 0.16). Repeated cocaine treatment induced no significant change in GluR2 levels (t(10) = 0.40, p = 0.69), suggesting that 7 days of exposure to cocaine increases synaptic expression of GluR1, but not pan-AMPA and GluR2, in the NAc of WT mice. Finally, the effects at accumbal synapses in β2m^−/−^ mice were also evaluated (Figs. 4D, 4F). The densities normalized to saline in each receptor were: pan-AMPA: β2m^−/−^-Coc, 130.9±10.0%; GluR1: β2m^−/−^-Coc, 117.5±5.8%; GluR2: β2m^−/−^-Coc, 119.7±6.2%, n = 6 [for each receptor 6 replicas from three animals, 30 synapses per replica]. Repeated cocaine administration induced a significant increase in the levels of pan-AMPA, GluR1 and GluR2 on synapses of β2m^−/−^ mice compared with repeated saline administration (pan-AMPA: t(10) = 2.28, p<0.05; GluR1: t(10) = 2.71, p<0.05; GluR2: t(10) = 2.93, p<0.05). This result indicates that β2m levels influence cocaine-induced AMPA receptor expression during synaptic adaptation.

## Discussion

In this study, we first found by immunohistochemical staining that MHCI molecules are expressed in NAc-core neurons in wild-type mice. This indicates that MHCI may have a role in modulation of synaptic plasticity in the NAc. To investigate this possibility, we analyzed β2m^−/−^ mice deficient of cell-surface expression of MHCI. We performed electrophysiological recordings from accumbal slices and found that high frequency stimulation induced LTP and low frequency stimulation induced LTD in WT mice. In contrast, in β2m^−/−^ mice, LTP was enhanced and LTD was not observed. Next, we found that β2m^−/−^ mice showed significantly higher behavioral sensitization than WT mice in response to repeated cocaine exposure. Finally, when we analyzed the densities of AMPA receptors and their subunits on excitatory synapses of NAc core, we found that repeated exposure to cocaine significantly increased only GluR1 expression in WT mice, whereas all pan-AMPA, GluR1 and GluR2 receptors showed significantly higher expression in β2m^−/−^ mice. Taken together, these findings demonstrate that β2m^−/−^ mice show clearly augmented synaptic potentiation induced by electrical and pharmacological stimulation in comparison with WT mice.

Mice deficient in MHCI function show enhanced LTP and abolished LTD in the hippocampus [9], [11], [12]. Consistent with these results, we found, for the first time, enhanced LTP and abolished LTD in the NAc of mice with deficient MHCI function. Thus, accumbal MHCI could regulate LTP and LTD through the same mechanism observed in the hippocampus. NMDA [41], endogenous cannabinoid [42], and metabotropic glutamate [43] receptors modulate the threshold for LTD in the hippocampus. Additionally, a recent study demonstrated that MHCI proteins are only critical for hippocampal NMDA receptor-dependent LTD [11]. Indeed, it’s already known that NMDA receptor function is enhanced in the hippocampus of MHCI-deficient mice (Fourgeaud et al., 2010), therefore the altered LTP and LTD in the NAc we observed in this study is also likely to be the result from functionally changed NMDA receptors in MHCI-deficient mice. Future studies will be necessary to decipher the mechanisms underlying the observed LTD changes by examining with pharmacological approaches that allow for the independent induction of receptor-dependent LTD using CNQX (MPA/kainate antagonist), AP5 (NMDA receptor antagonist), LY341495 (mGluR2/3 antagonist) or SR141716 (CB1 antagonist) (Kombian and Malenka, 1994; Robbe et al., 2002; Nelson et al., 2013).

As mentioned above, for the first time, we report that β2m^−/−^ mice show greater behavioral sensitization than WT mice following repeated cocaine injections. One important brain area underlying behavioral sensitization is the NAc, and glutamatergic efferents from prefrontal cortex to NAc core are critical for this form of sensitization (Kalivas, 2009). In our SDS-FRL experiments, the density of the AMPA receptors, which is related to the strength of synaptic connection, was increased in WT mice repeatedly treated with cocaine, and this result is consistent with the above-mentioned report (Kalivas, 2009). In WT mice, we found that repeated cocaine exposure significantly increased the density of GluR1 on synapses of neurons in the NAc. A previous study by Boudreau and Wolf (2005) reported that repeated cocaine exposure increased cell-surface expression of not only GluR1 but also GluR2/3 in the NAc following 10–21 days of withdrawal. Although the protein cross-linking assay used in this report can distinguish between cell surface and intracellular AMPA receptor, the method cannot discriminate extra-synaptic receptors from synaptic receptors that are directly involved in synaptic transmission. We directly analyzed the density of AMPA receptor in the synapse and found that only GluR1 expression was increased in synapses. Interestingly, in β2m^−/−^ mice repeatedly treated with cocaine, the densities of accumbal pan-AMPA, GluR1 and GluR2 on excitatory synapses were all higher than in saline-treated animals. In a previous study using primary culture of hippocampal neurons, immunostaining revealed significantly increased surface GluR1 in the neurons of MHCI-deficient mice, but not WT mice, and there was no significant change in GluR2 in either genotype, shortly following NMDA treatment [44]. Although our results are not exactly consistent with those garnered using immunostaining if we focus on the changes of each subunit, at least we would be able to say that AMPA receptor subunits tend to increase in MHCI-deficient mice after stimulating NMDA receptors. The increased density of AMPA receptors in the NAc we observed, therefore, may have resulted from the loss of MHCI regulation of AMPA receptor trafficking via NMDA receptors.

In this study, we did not evaluate any effects of an impaired immune system itself on the parameters measured here. Therefore we should be careful in our interpretation that the changes observed in β2m^−/−^ mice purely resulted from the absence of β2m in the nucleus accumbens. Recently, Lee et al. [45] performed an elegant experiment to determine the contribution of the immune system and to examine whether neuronal expression of MHCI is sufficient for normal synaptic plasticity, by using H2-K^b^D^b^ knock-out (H2-K^b^D^b−/−^) mice. By crossing these mice with NSED^b+^ mice in which H2-D^b^ expression is regulated under the neuron-specific enolase (NSE) promoter, Lee et al. rescued their H2-D^b^ expression exclusively to neurons while H2-K^b^D^b−/−^ remains in the rest of the body. As a result, they found that neuronal H2-D^b^ expression was enough for rescuing normal synaptic plasticity. This would be one supportive example that impaired immune system in the body has no effect on synaptic plasticity in the brain.

In conclusion, we found that MHCI proteins are expressed in the NAc of WT mice. We also found that LTP is enhanced whereas LTD is abolished in functional MHCI-deficient mice. We finally observed that baseline of AMPA receptor expression is normal, but there is an increase in AMPA receptor density including both GluR1 and GluR2 following repeated cocaine administration in functional MHCI-deficient mice, whereas only GluR1 is increased in WT mice. Thus, the present study indicates that MHCI proteins may play a role in lowering the strength of synaptic connections in the NAc. Furthermore, an interesting study has recently reported the persistence of abolished LTD in rats that displayed typical addiction-like behavior [46]. Our new findings may offer insights into possible mechanistic basis for vulnerability to addiction as altered expression levels of MHCI molecules at NAc synapses may trigger changes in synaptic structure associated with the transition to addiction, generating long-lasting alteration in LTD underlying addiction-like behavioral adaptations.
